# Auto-segmentation of Adult-Type Diffuse Gliomas: Comparison of Transfer Learning-Based Convolutional Neural Network Model vs. Radiologists

**DOI:** 10.1007/s10278-024-01044-7

**Published:** 2024-02-21

**Authors:** Qi Wan, Jisoo Kim, Clifford Lindsay, Xin Chen, Jing Li, J. Bryan Iorgulescu, Raymond Y. Huang, Chenxi Zhang, David Reardon, Geoffrey S. Young, Lei Qin

**Affiliations:** 1 Department of Imaging, Dana Farber Cancer Institute, Harvard Medical School, Boston, MA USA; 2 Department of Radiology, the Key Laboratory of Advanced Interdisciplinary Studies Center, the First Affiliated Hospital of Guangzhou Medical University, Guangzhou, China; 3 Department of Radiology, Brigham and Women’s Hospital, Harvard Medical School, Boston, MA USA; 4 Image Processing and Analysis Core (iPAC), Department of Radiology, University of Massachusetts Chan Medical School, Worcester, MA USA; 5 School of Medicine, Guangzhou First People’s Hospital, South China University of Technology, Guangzhou, Guangdong China; 6 Department of Radiology, the Affiliated Cancer Hospital of Zhengzhou University (Henan Cancer Hospital), Zhengzhou, China; 7 Molecular Diagnostics Laboratory, Department of Hematopathology, Division of Pathology and Laboratory Medicine, The University of Texas MD Anderson Cancer Center, Houston, USA; 8 Digital Medical Research Center, School of Basic Medical Sciences, Fudan University, Shanghai, China; 9 Center for Neuro-Oncology, Dana-Farber Cancer Institute, Boston, MA USA

**Keywords:** Glioma, BraTS, Deep learning, Segmentation

## Abstract

Segmentation of glioma is crucial for quantitative brain tumor assessment, to guide therapeutic research and clinical management, but very time-consuming. Fully automated tools for the segmentation of multi-sequence MRI are needed. We developed and pretrained a deep learning (DL) model using publicly available datasets A (*n* = 210) and B (*n* = 369) containing FLAIR, T2WI, and contrast-enhanced (CE)-T1WI. This was then fine-tuned with our institutional dataset (*n* = 197) containing ADC, T2WI, and CE-T1WI, manually annotated by radiologists, and split into training (*n* = 100) and testing (*n* = 97) sets. The Dice similarity coefficient (DSC) was used to compare model outputs and manual labels. A third independent radiologist assessed segmentation quality on a semi-quantitative 5-scale score. Differences in DSC between new and recurrent gliomas, and between uni or multifocal gliomas were analyzed using the Mann–Whitney test. Semi-quantitative analyses were compared using the chi-square test. We found that there was good agreement between segmentations from the fine-tuned DL model and ground truth manual segmentations (median DSC: 0.729, std-dev: 0.134). DSC was higher for newly diagnosed (0.807) than recurrent (0.698) (*p* < 0.001), and higher for unifocal (0.747) than multi-focal (0.613) cases (*p* = 0.001). Semi-quantitative scores of DL and manual segmentation were not significantly different (mean: 3.567 vs. 3.639; 93.8% vs. 97.9% scoring ≥ 3, *p* = 0.107). In conclusion, the proposed transfer learning DL performed similarly to human radiologists in glioma segmentation on both structural and ADC sequences. Further improvement in segmenting challenging postoperative and multifocal glioma cases is needed.

## Introduction

Adult-type diffuse gliomas represent a heterogeneous group of primary central nervous system (CNS) tumors with distinct molecular profiles, clinical behavior, and prognosis [1]. Segmentation of structural and physiologic/functional magnetic resonance imaging (MRI) sequences will be critical if recent advances in quantitative MRI assessment are to be translated to clinical research and care [2]. Quantification of changing tumor size, contrast enhancement, and cellularity over time have been demonstrated to increase the precision of treatment monitoring in clinical trials, and advanced image analysis techniques including radiomics, have demonstrated promise in glioma grading, molecular characterization, and survival prediction [3–5]. However, manual segmentation of gliomas by radiologists is time-intensive and prone to intra- and inter-observer variability [6, 7].

Convolutional neural networks (CNNs) have recently been demonstrated to improve the accuracy and efficiency of brain tumor segmentation [8–10]. Direct training of CNN using multimodal volumetric brain MRI requires prohibitively large amounts of manually labeled ground truth data and extensive computational resources, and is susceptible to overfitting when applied to smaller datasets [11]. Transfer learning enables researchers to adapt deep learning (DL) models pre-trained for image analysis using existing large labeled external sets of similar image data, enabling the creation of effective MRI brain tumor segmentation DL models without the need for large, labeled local ground truth datasets.

The publicly available Brain Tumor Segmentation (BraTS) database consists of multimodal brain MRI including T1, T2, FLAIR-T2, and contrast-enhanced T1 (T1CE)-weighted images. Segmentation algorithms developed using BraTS data have reported improving segmentation performance in recent years [12], but clinical application remains limited. In addition to technical barriers to implementation, most previous studies have focused on preoperative imaging, whereas the most important potential clinical use would be sensitive detection of longitudinal change during postoperative treatment. Also, generalizability, stability, and robustness in clinical use remain to be validated [13]. Finally, although the value of diffusion-weighted imaging (DWI) and quantitative apparent diffusion coefficient (ADC) has been established to improve initial evaluation and posttreatment assessment of brain tumors [14], with evidence showing that including DWI and/or ADC improves performance of radiomic models for tumor grading, prediction of IDH-mutation-status [15] and identification of pseudo-progression [16], automatic segmentation of DWI and/or ADC have seldom been reported.

We report the development and validation of a transfer learning-based automatic segmentation DL model for segmenting structural (T1CE, FLAIR) and physiologic/functional (ADC) brain MRI. Pretraining with 2 publicly available BraTS datasets with conventional sequences and subsequent fine-tuning on local radiologist-annotated real-world MRI data including ADC maps ensured a diverse set of MR images with well-annotated masks. Quantitative and qualitative evaluation on an isolated local MRI test set demonstrated performance comparable to radiologist segmentations.

## Method

**Fig. 1:**
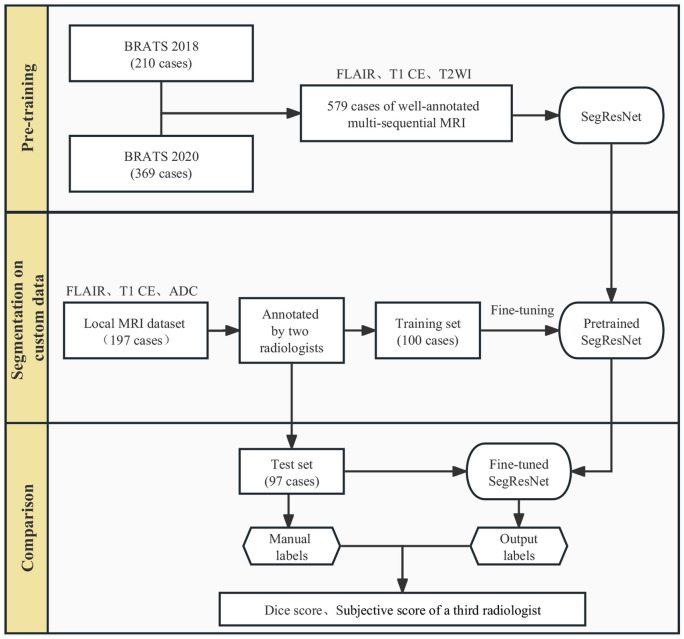
Flowchart of this study

This HIPAA-compliant, retrospective medical records study was approved by our institutional IRB. Figure 1 illustrates the 3 workflow stages: pre-training, fine-tuning and evaluation.

### Dataset

We used the BraTS 2018[17–19] (*n* = 210) and BraTS 2020[17–19] (*n* = 369) datasets for pretraining our model. Our institutional data included 197 patients with histopathologically confirmed adult-type diffuse gliomas treated at our center from April 2014 to February 2019 (Table 1). This very heterogeneous, chronologically selected patient cohort received immune checkpoint inhibitors and underwent serial clinical MRI on 12 different scanners from two vendors (GE Medical Systems^®^ and Siemens Healthineers^®^). Our data was randomly divided into training (*n* = 90), validation (*n* = 10), and test sets (*n* = 97).

**Table 1:** Characteristics of our patient cohort

**Pathology**	**Grade**	**IDH status**	**Number of patients (*n* = 197)**	**Age**
Glioblastoma	4	IDH-wt	176	57.7 ± 10.8
Astrocytoma	2-4	IDH-mut	19	39.4 ± 13.9
Oligodendroglioma (1p/19q co-deletion)	3	IDH-mut	1	51
Diffuse midline glioma (H3K27M-altered)	4	IDH-wt	1	45

### Image Preprocessing

BraTS multimodal scans consisted of four sequences (T1WI, T2WI, FLAIR-T2, and T1CE), All images were skull-stripped, co-registered to the same anatomical frame of reference, and interpolated to the same resolution (1 × 1 × 1 mm^3^). From our institutional data, we selected three sequences (FLAIR-T2, T1CE, and ADC). ADC and FLAIR-T2 were co-registered to T1CE and resampled to match the original resolution of the T1CE (1 × 1 × 1 mm^3^). To match our 3-channel institutional data, we selected three sequences from the BraTS data (T2WI, FLAIR-T2, T1CE) and stacked them to create a three-channel input.

### Tumor Annotation

Annotations included in the BraTS datasets consisted of three sub-compartmental segmentation masks: tumor core (TC), whole tumor (WT), and enhancing tumor (ET). Two radiologists (with 10 years and 7 years of experience respectively) manually annotated our local data to select 3D ROIs on each of the three sequences (FLAIR-T2, T1CE, and ADC) using 3D Slicer^®^ (Harvard Medical School, Boston, MA, USA, https://www.slicer.org) [20], with each radiologist annotating a portion of the dataset. These annotations included three segmentation labels matching the BraTS dataset: label “1” representing the enhancing tumor; label “2” representing the abnormal ADC area, which includes both the edema with high ADC values and the tumor with intermediate ADC values; label “3” representing the area of hyperintensity on the FLAIR-T2 images. Label “0” representing the background image not included in any of the other masks. These 4 labels were stacked to construct a corresponding set of masks.

### Deep Learning Model

We used the SegResNet [21] CNN, with the Adam optimizer, a learning rate of 1e^−4^ and cosine annealing as the scheduler. We combined DiceLoss and CrossEntropy Loss as the loss functions for optimizing the model’s performance.

### Quantitative Evaluation

We evaluated the model performance on our datasets using the Dice similarity coefficient (DSC), which is commonly used to evaluate segmentation [22] and offers a quantitative measure of similarity between the predicted and manual segmentations. It was calculated using the formula:

DSC = 2 * (area of overlap of both segmentations)/(total area of both segmentations).

The value ranges from 0 to 1, with a higher DSC indicating better segmentation performance relative to the gold standard. DSC > 0.7 is generally considered to indicate good performance [23].

### Model Training Process

We allocated 464 studies for training and 115 studies for validation (8:2 training: validation ratio). The training epoch was set to 100. At epoch 92, the model achieved the highest average DSC on the validation data. The DSC for the different tumor sub-compartments were as follows: TC at 0.8684, WT at 0.8963, ET at 0.8214, and an overall average of 0.862. After pretraining, we fine-tuned the model using our private training and validation dataset (90 training and 10 validation cases) for 50 epochs, with the same optimizer, learning rate, and loss functions as used for pretraining. The DSC of our final model on our clinical validation data were as follows: WT at 0.7924, ET at 0.6789 and ADC at 0.5813.

### Semi-quantitative Evaluation

**Fig. 2:**
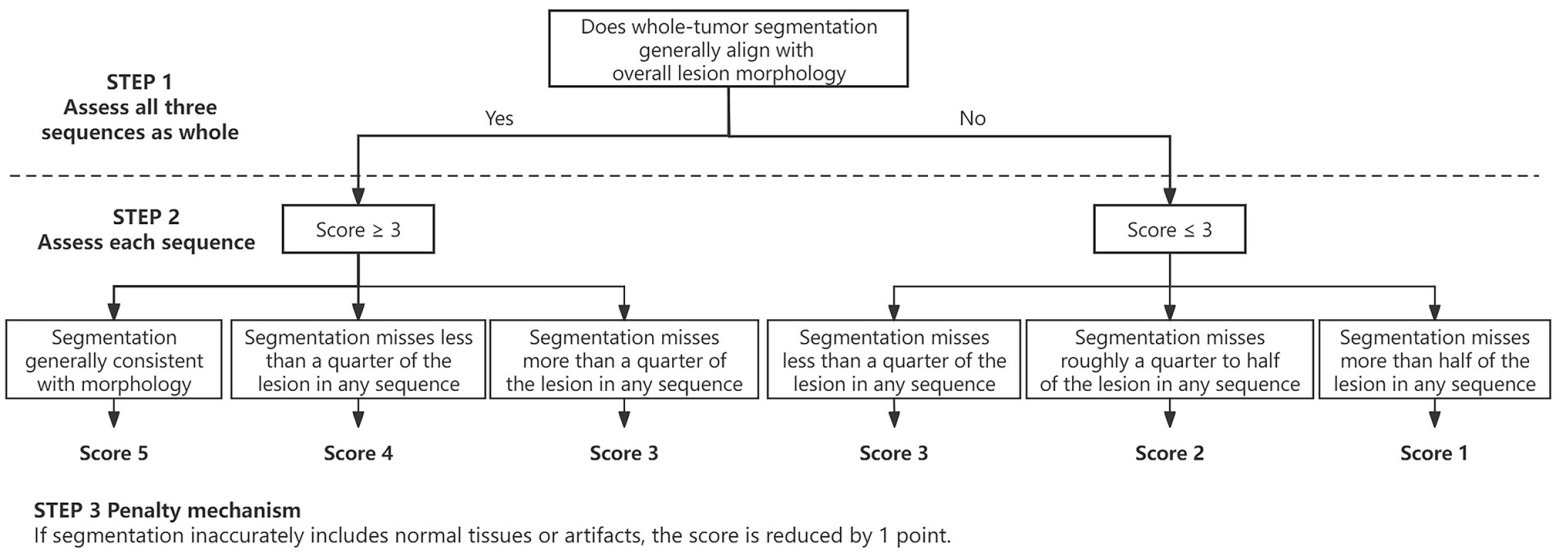
Flowchart of two-step 5-point scale assessment for evaluating segmentation quality

To evaluate the segmentation quality of our institutional dataset, we introduced an additional semi-quantitative five-point scale, where the ratings are defined as 5 (“Excellent”), 4 (“Very Good”), 3 (“Good”), 2 (“Fair”), and 1 (“Poor”). The flowchart of the assessment is illustrated in Fig. 2.

Initially, segmentations are reviewed for general consistency with the lesion’s morphology across all sequences. If it is generally consistent with lesion morphology, the evaluation advances to the second step with an initial score of 3 or higher. Otherwise, the segmentation is deemed inconsistent, and is given an initial score of 3 or lower.

In the second step, a detailed sequency-by-sequence review is conducted. For segmentations initially scored 3 or higher, the scale is as follows: A score of 5 is given for complete consistency across all sequences. A score of 4 applies if a visual estimate indicates less than a quarter of the tumor volume is missing in any sequence. A score of 3 is assigned if it appears more than a quarter is missing in any sequence. For segmentations initially scored at 3 or lower: A score of 3 is maintained if less than a quarter is visually missing in any sequence. A score of 2 is given when an estimated quarter to a half of the tumor volume is missing in any sequence. A score of 1 is reserved for cases where more than half of the tumor volume appears to be missing. Additionally, a penalty of 1 point is applied to any segmentation erroneously including normal anatomy or artifacts.

To quantify the inter-rater reliability of these ordinal ratings, we calculated the Intraclass Correlation Coefficient (ICC) using a two-way mixed model for consistency based on independent reviews of 30 randomly selected cases by two radiologists (with 10 and 7 years of experience, respectively). Finally, the semi-quantitative evaluations on all cases were done by the radiologist with 10 years of experience.

### Statistical Analysis

The DSC of different groups or subgroups was compared using the Mann–Whitney *U* test. The semi-quantitative scores of different groups or subgroups were compared using the Chi-squared test. Statistical analyses were performed using SPSS Statistics for Windows, (Version 22.0, IBM Corp.). A two-sided p-value of < 0.05 was considered significant.

## Results

### Quantitative Evaluation

In our local 97 glioma test set, 74 were unifocal lesions and 23 were multifocal; 16 were MRI at the time of initial diagnosis and 81 were MRI performed at the time of recurrence. Using the fine-tuned DL model, we achieved a median DSC of 0.729 with a standard deviation of 0.134 for the whole tumor mask, demonstrating good agreement between the DL and manual segmentations.

The DSC of DL model was superior for newly diagnosed cases (median (P25–P75): 0.807 (0.785–0.832) compared to recurrent cases (median (P25–P75): 0.698 (0.558–0.772), *p* < 0.001, and superior for unifocal cases (median (P25–P75): 0.747 (0.813–0.655) compared to multifocal cases (median (P25–P75): 0.613 (0.475–0.758), *p* = 0.001.

### Semi-quantitative Assessment

**Fig. 3:**
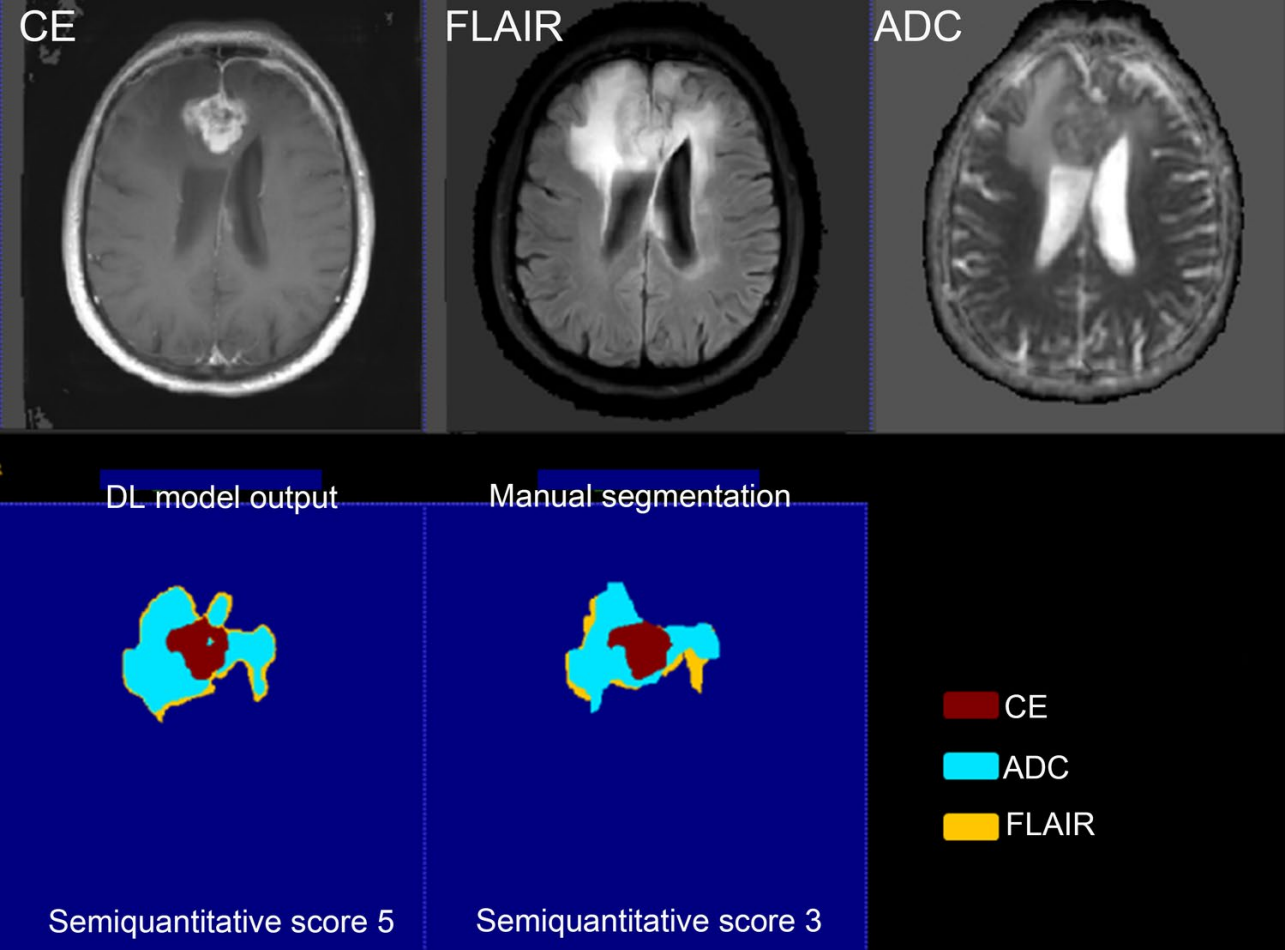
A representative case for manual and deep learning segmentation. Dice coefficient is 0.816. Semi-quantitative score: DL segmentation is consistent with tumor morphology overall (score ≥ 3); individual sequence analysis shows uniform consistency across all sequences, meriting a score of 5. Manual segmentation is inconsistent with overall tumor morphology (score ≤ 3); ADC and FLAIR sequences specifically exhibit roughly less than a quarter missing volume, leading to a score of 3

**Fig. 4:**
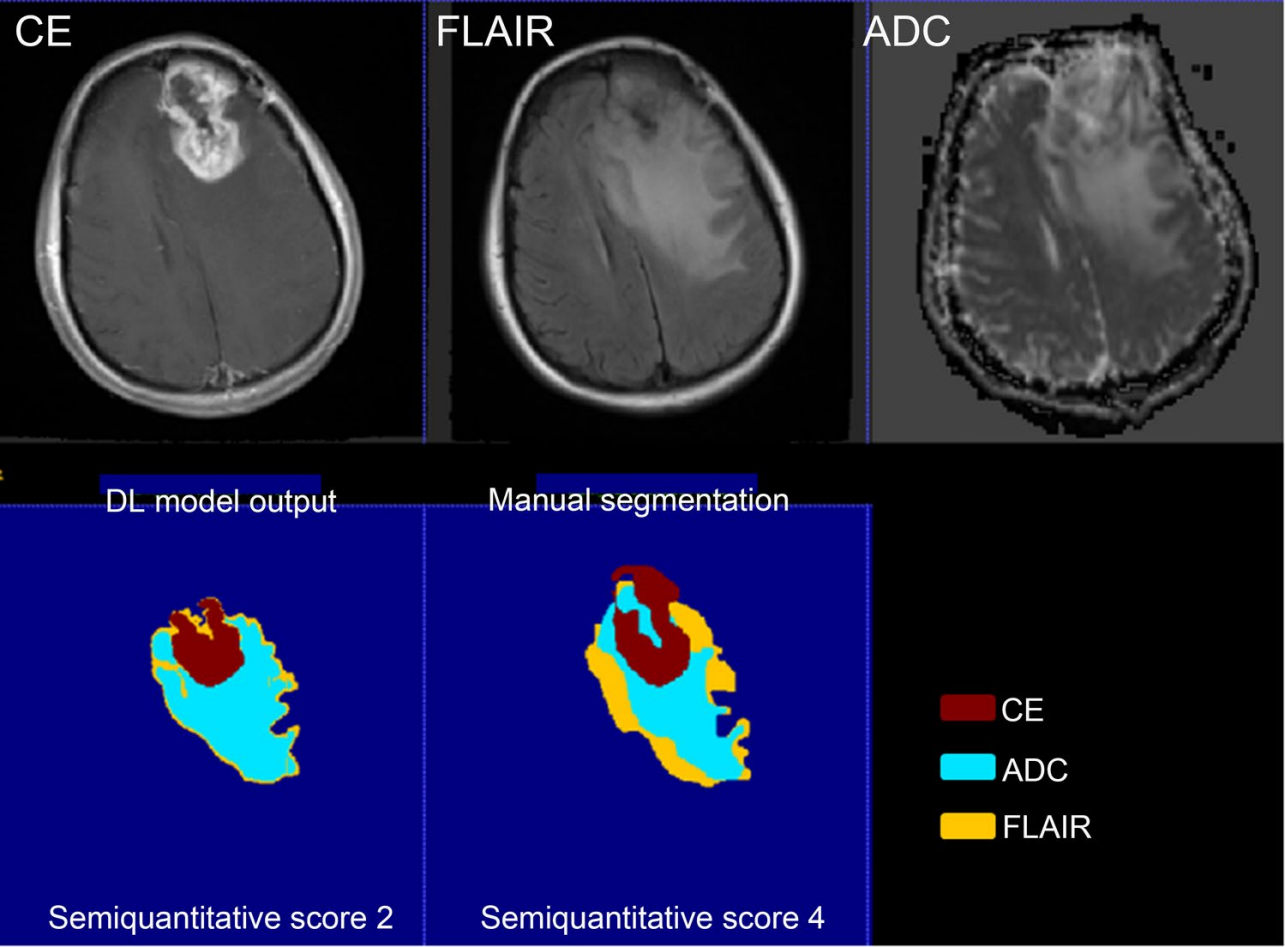
A representative case for manual and deep learning segmentation. Dice coefficient is 0.7636. Semi-quantitative score: DL segmentation is not consistent with overall tumor morphology (score ≤ 3); detailed analysis shows roughly a quarter to half missing in CE sequence, leading to a score of 2. Manual segmentation is generally consistent with tumor morphology (score ≥ 3); sequence-by-sequence assessment reveals less than a quarter missing in ADC sequence, resulting in a score of 4

The ICCs between the two radiologists for assessing manual and deep learning segmentation were 0.759 and 0.770 respectively. Table 2 presents the semi-quantitative scores from all test cases. The average semi-quantitative score for DL segmentations was 3.639 ± 0.710, compared to 3.567 ± 0.789 for manual segmentation. In the DL group, 93.8% of the scores were 3 or higher, compared to 97.9% in the manual segmentation group. No statistically significant difference was observed between the two groups (*p* = 0.107). Two representative cases of DL and corresponding segmentation are shown in Fig. 3 and Fig. 4.

**Table 2:** The semiquantitative scores of DL and manual segmentations

**Semiquantitative score**		**Manual segmentation (%)**				**Total**	***P* value**
		**2.0**	**3.0**	**4.0**	**5.0**		
**Deep learning**	**2.0**	0 (0.00)	1 (2.38)	5 (11.90)	0 (0.00)	6 (6.19)	0.107
	**3.0**	0 (0.00)	23 (54.76)	17 (40.48)	2 (18.18)	42 (43.30)	
	**4.0**	2 (100.00)	15 (35.71)	13 (30.95)	7 (63.64)	37 (38.14)	
	**5.0**	0 (0.00)	3 (7.14)	7 (16.67)	2 (18.18)	12 (12.37)	
**Total**		2	42	42	11	97

Subgroup analysis of the semi-quantitative scores (Table 3) revealed no performance differences between newly diagnosed and recurrent gliomas, or between unifocal and multifocal gliomas for the DL model (*p* = 0.367 and 0.357 respectively). Analysis of manual segmentation did not reveal performance differences between newly diagnosed and recurrent gliomas (*p* = 0.315), but manual segmentation performed better for unifocal than multifocal glioma (*p* = 0.015).

**Table 3:** The subgroup analysis of semi-quantitative scores

**Method**	**Semi-quantitative score**	**Single (%)**	**Multiple (%)**	**P value**	**Newly (%)**	**Recurrent (%)**	***P* value**
Deep learning	**2**	3 (4.05)	3 (13.04)	0.357	0 (0.00)	6 (7.41)	0.367
	**3**	31 (41.89)	11 (47.83)		5 (31.25)	37 (45.68)	
	**4**	30 (40.54)	7 (30.43)		8 (50.00)	29 (35.80)	
	**5**	10 (13.51)	2 (8.70)		3 (18.75)	9 (11.11)	
	**Total**	74	23		16	81	
Manual	**2**	0 (0.00)	2 (8.70)	0.015*	0 (0.00)	2 (2.47)	0.315
	**3**	30 (40.54)	12 (52.17)		4 (25.00)	38 (46.91)	
	**4**	33 (44.59)	9 (39.13)		9 (56.25)	33 (40.74)	
	**5**	11 (14.86)	0 (0.00)		3 (18.75)	8 (9.88)	
	**Total**	74	23		16	81	

^*^denotes *p* < 0.05

### Failure Analysis of DL Segmentation

Our analysis revealed two main situations where the DL segmentation performs poorly with a DSC below 0.3.

**Fig. 5:**
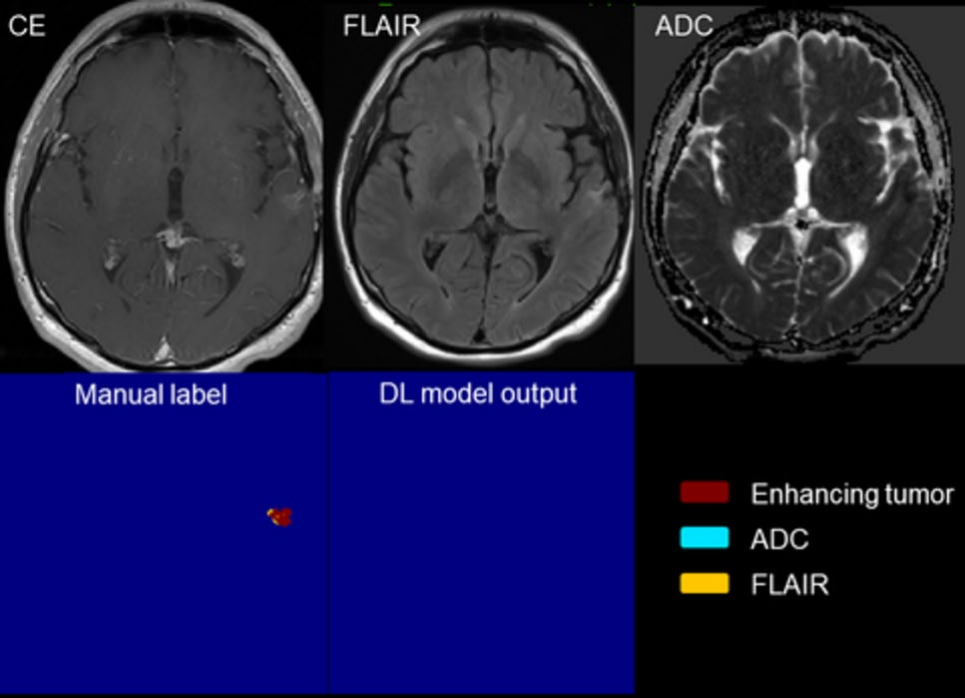
A recurrent tumor. This figure depicts a small, recurrent tumor characterized by faint enhancement. The DL algorithm failed to label this region

The first is on segmenting post-surgery tumor, illustrated in Fig. 5. Here, the enhancing tumor is located within a surgical resection cavity. This unusual presentation differs markedly from typical glioma cases, making it challenging for the model to correctly identify the tumor boundaries. This reflects the difficulties faced by the DL model in post-surgical situations.

**Fig. 6:**
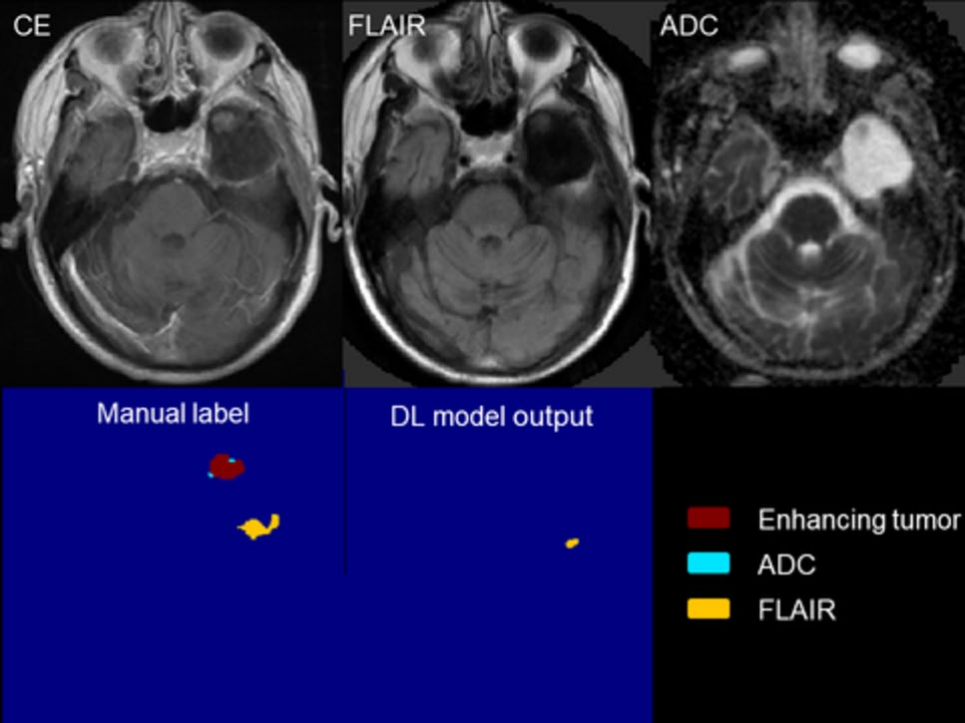
A postoperative recurrent tumor. Illustrated here is an enhancing tumor situated within a post-surgical resection cavity. The DL algorithm failed to label this region

The second is on segmenting small or faintly enhancing tumor, illustrated in Fig. 6, where a small, recurrent tumor with only slight enhancement was shown in the image. The tumor’s small size and low contrast enhancement posed significant challenges for the model, impairing its ability to detect and segment the tumor effectively. This underscores the limitations of the DL model in accurately identifying small tumors with subtle imaging features.

## Discussion

We developed and evaluated a transfer learning-based automatic tumor segmentation DL model using multi-sequential MRI data. Our results suggest that the DL segmentation model’s performance on our real-world MRI data is comparable to that of manual segmentation, as evidenced by semi-quantitative evaluation from an independent radiologist. Although pretrained on large datasets with structural imaging only, the model proved effective in the automatic segmentation of both structural MRI and ADC maps, demonstrating substantial robustness.

Regarding the challenge of evaluating glioma segmentation performance in clinical situations, where ground truth labels may be imperfect or unavailable, our study contributes by introducing a 5-point semi-quantitative scale, offering a practical tool with proven reproducibility for radiologists and researchers to assess segmentation quality. We noted that certain DL cases with suboptimal DSC scores received higher subjective evaluations. This observation underscores the importance of including semi-quantitative assessment in the analysis of DL model performance. We suggest that the combination may offer a more meaningful and valid assessment of output quality than DSC alone. This is particularly relevant when using local institutional datasets, that may not be as accurately segmented as established publicly available datasets like BraTS, because of the shortage of radiologists [24], demanding radiological workflow and time required for manual segmentation. Although experienced radiologists can quickly determine which segmentation is superior, it is far more time-consuming to actually produce precise detailed manual segmentations. This raises the possibility that the DSC of 0.6–0.8 may in part be due to inaccuracies in the 3D ROIs used as a ground truth and hence may lead to underestimation of the true performance of the DL model. Furthermore, the absence of DWI or ADC data in the initial publicly available training sets, and the relatively limited size of our institutional training dataset are two other factors that may have contributed to the less-than-ideal DSC scores. We speculate that geometric distortion characteristic of the echo planar DWI sequence and zero-value pixels present in the calculated ADC maps might pose challenges for a transfer learning model trained on data lacking echo planar images or calculated maps.

A range of methodologies has been reported for brain tumor segmentation, yielding varied outcomes. Singh et al. [25] applied a 3D U-Net with transfer learning across multiple BraTS datasets, achieving a notable mean DSC of 0.98. However, their study lacks external validation with real-world data. Another study [11] evaluated two AlbuNet3D models on the BraTS dataset: one model underwent pretraining on Imagenet, followed by transfer learning on the BraTS data, while the other was trained directly on BraTS data. Notably, the transfer learning model, although showing enhanced performance on the BraTS dataset, did not exhibit a significant improvement over the directly trained model when applied to a more heterogeneous, local institution clinical dataset. This outcome may partly be attributed to the difference in the data distribution and characteristics between the BraTS dataset and the local clinical dataset. The BraTS dataset is a standardized, curated collection of brain tumor images, which may not fully represent the variability and complexity encountered in routine clinical practice. In BraTS challenge, the nnU-Net [26] demonstrated remarkable success, achieving the first place in the BraTS 2020 [27] and 2021 [28] with DSCs of 0.889 and 0.931 for whole tumor, respectively. A recent study [29] used nnU-Net on local clinical data resulted in a performance drop (0.906 on BraTS vs 0.764 for WT on local data), similar to our findings (0.896 vs 0.729) with more heterogeneous and larger test data. This underscores the inherent challenge in maintaining model accuracy outside standardized datasets. Bouget et al. [30] developed an open-source automatic glioblastoma segmentation tool using nnU-Net. It demonstrated considerable robustness across 14 different MRI data sources, achieving an average DSC of 0.866, but exclusively relied on T1CE MRI sequences and did not include postoperative or recurrent tumor cases. These factors constrain its broader clinical utility, especially in GBM response assessment where evaluation of both enhancing and non-enhancing tumor components is critical. Our study demonstrates that transfer learning, refined with a small private dataset, can yield effective segmentation models, rivaling manual segmentation in accuracy in not only structural sequences (FLAIR, CE) but also functional sequence (ADC).

Our institutional MRI data is both technically and clinically more heterogeneous than the BraTS training set, including more complicated cases, both pre- and postoperative, single and multiple lesions, and multiple MRI sequences including ADC. Consistent with the prior literature, the DL model achieved higher DSC in segmenting newly diagnosed glioma cases compared to recurrent cases [31, 32]. Training dedicated models for postoperative treatment monitoring may achieve better performance and is a logical next step.

The semi-quantitative assessment subgroup analysis showed higher quality of manual segmentation in unifocal vs multifocal GBM, but DL segmentation quality was not rated as significantly different between newly diagnosed and recurrent gliomas, or between unifocal and multifocal gliomas. Multifocal gliomas are more challenging to manually segment because of the complex shape and additional time required. As discussed, this raises the possibility that variation in the manual gold standard may contribute to variation in the quantitative DSC. Alternatively, it is possible that the semi-quantitative analysis was not sufficiently sensitive to detect an underlying decrement in DL segmentation that also contributed to the lower DSC in complex cases. This underscores the potential benefit of integrating both approaches to offer a more comprehensive assessment of segmentation quality.

Limitations of the study include the very heterogeneous dataset inherent to a multi-center, retrospective study, and the small sample size of our cohort. These constraints might be mitigated, at least in part, by training models on larger datasets specifically focusing on complex populations such as postoperative cases, if such datasets become available. Second, more sophisticated DL models than that we used have recently become available, raising the possibility that our results could be further improved by using a more advanced structure, such as Swin UNETR [33]. Because these technically more sophisticated models require more data and computational power than we were able to achieve, we chose the reported method as a balance between computational demands and model performance. Lastly, our ADC annotations encompass both tumor and edema regions, while in clinical practice, annotations often focus on intermediate ADC regions that indicate hypercellular tumor zones and are predictive of outcome. We chose this approach because our primary objective was to demonstrate the potential of transfer learning-based DL model for segmentation of the overall area of abnormality on ADC maps, since, as has been previously demonstrated, the auto-normalized quantitative nature of the ADC maps makes it straightforward to identify the smaller intermediate ADC area by simple thresholding [34].

In conclusion, our study introduces a 5-point semi-quantitative scale that offers a reliable reference, simplifying assessments of glioma segmentation in clinical situations for radiologists and researchers. Our study demonstrates that a transfer learning-based DL model, fine-tuned on a relatively small private dataset, produces comparable performance to that of human radiologists in glioma segmentation on both structural and functional data. This approach has the potential to improve longitudinal imaging assessment of treatment response in clinical care and clinical trials. Future research should focus on optimizing the DL model for challenging clinical applications and patient populations, especially postoperative recurrent glioma and multifocal glioma, as well as assessing the generalizability of model performance on larger, multi-center external datasets, and the stability of performance over time in longitudinal prospective use.

## Data Availability

The data are not publicly available due to privacy or ethical restrictions.
